# Highly focused transcriptional response of *Anopheles coluzzii* to O’nyong nyong arbovirus during the primary midgut infection

**DOI:** 10.1186/s12864-018-4918-0

**Published:** 2018-07-09

**Authors:** Guillaume Carissimo, Adrien Pain, Eugeni Belda, Kenneth D. Vernick

**Affiliations:** 1 Unit of Insect Vector Genetics and Genomics, Department of Parasites and Insect Vectors, Institut Pasteur, Paris, France; 2 CNRS Unit of Evolutionary Genomics, Modeling, and Health (UMR2000), Institut Pasteur, Paris, France; 3 Laboratory of Microbial Immunity, Singapore Immunology Network, Agency for Science, Technology and Research (A(∗)STAR), Singapore, Singapore; 4 Bioinformatics and Biostatistics Hub (C3BI), USR 3756 IP CNRS, Institut Pasteur, 75017 Paris, France; 5 Integromics Unit, Institute of Cardiometabolism and Nutrition, Assistance Publique Hôpitaux de Paris, Pitié-Salpêtrière Hospital, Paris, France

**Keywords:** Arbovirus, Insect vectors, Insect immunity, Host–pathogen interactions, Innate immunity, Malaria

## Abstract

**Background:**

*Anopheles* mosquitoes are efficient vectors of human malaria, but it is unknown why they do not transmit viruses as well as *Aedes* and *Culex* mosquitoes. The only arbovirus known to be consistently transmitted by *Anopheles* mosquitoes is O’nyong nyong virus (ONNV, genus Alphavirus, family Togaviridae). The interaction of *Anopheles* mosquitoes with RNA viruses has been relatively unexamined.

**Results:**

We transcriptionally profiled the African malaria vector, *Anopheles coluzzii,* infected with ONNV. Mosquitoes were fed on an infectious bloodmeal and were analyzed by Illumina RNAseq at 3 days post-bloodmeal during the primary virus infection of the midgut epithelium, before systemic dissemination. Virus infection triggers transcriptional regulation of just 30 host candidate genes. Most of the regulated candidate genes are novel, without known function. Of the known genes, a significant cluster includes candidates with predicted involvement in carbohydrate metabolism. Two candidate genes encoding leucine-rich repeat immune (LRIM) factors point to possible involvement of immune protein complexes in the mosquito antiviral response. The primary ONNV infection by bloodmeal shares little transcriptional response in common with ONNV infection by intrathoracic injection, nor with midgut infection by the malaria parasites, *Plasmodium falciparum* or *P. berghei*. Profiling of *A. coluzzii* microRNA (miRNA) identified 118 known miRNAs and 182 potential novel miRNA candidates, with just one miRNA regulated by ONNV infection. This miRNA was not regulated by other previously reported treatments, and may be virus specific. Coexpression analysis of miRNA abundance and messenger RNA expression revealed discrete clusters of genes regulated by Imd and JAK/STAT, immune signaling pathways that are protective against ONNV in the primary infection.

**Conclusions:**

ONNV infection of the *A. coluzzii* midgut triggers a remarkably limited gene regulation program of mostly novel candidate genes, which likely includes host genes deployed for antiviral defense, as well as genes manipulated by the virus to facilitate infection. Functional dissection of the ONNV-response candidate genes is expected to generate novel insight into the mechanisms of virus-vector interaction.

**Electronic supplementary material:**

The online version of this article (10.1186/s12864-018-4918-0) contains supplementary material, which is available to authorized users.

## Background

O’nyong-nyong virus (ONNV) and Chikungunya virus (CHIKV) are closely related alphaviruses of the Semliki Forest virus complex [1]. Both viruses cause febrile illness in humans and the symptoms are hard to distinguish from other alphavirus infections, Dengue fever or malaria [2]. Therefore, the ONNV burden on human populations has likely been underestimated due to misdiagnosis.

Indeed, a recent serodiagnostic study showed that in coastal Kenya, both CHIKV and ONNV circulate at high levels in human populations [3], with a high possibility of co-infections. *Anopheles* mosquitoes transmit ONNV, while CHIKV is transmitted by *Aedes* mosquitoes. More research effort has been focused on *Aedes* mosquitoes, due to their ability to also transmit multiple arboviruses such as dengue or Zika (Flaviviruses), which has led to a relative neglect about *Anopheles* interactions with arboviruses.

Virus survival of the bloodmeal digestive environment and initial infection of the midgut epithelium is thought to be the first infection bottleneck, called the midgut infection barrier (MIB) [4, 5]. The MIB is probably a distinct step from virus escape into the systemic compartment (the midgut escape barrier, MEB), followed by dissemination to other tissues including the salivary glands. Consistent with that prediction, we previously identified distinct antiviral immune signaling responses of the *Anopheles* primary midgut infection by bloodmeal, as compared to the subsequent systemic disseminated infection [6]. Earlier studies in which the virus was introduced by intra-thoracic injection [7–10] modeled only the disseminated systemic infection, even if it includes infection of midgut cells by dissemination, and do not measure the primary antiviral responses of the midgut.

To deepen the understanding of *Anopheles* interactions with RNA viruses, we sequenced messenger RNAs and small RNAs of *A. coluzzii* midguts three days after oral infection with ONNV, a time when the virus has not yet escaped to the systemic compartment [6]. Analysis of the datasets identified differentially regulated transcripts for messenger RNA (mRNA) and microRNA (miRNA) during ONNV infection of *A. coluzzii*, and predicted novel potential miRNAs. Transcriptional responses of the primary midgut ONNV infection were compared to published transcriptional datasets of *A. coluzzii* infected with ONNV by needle injection, and to the primary midgut infection with the eukaryotic protozoan malaria parasite, *Plasmodium*. In addition, joint analysis of mRNA and miRNA expression levels predicted putative regulatory networks of immune signaling.

## Results

### Differential *A. coluzzii* mRNA transcript abundance during ONNV infection

The primary infection of ONNV in the mosquito midgut after an infectious bloodmeal is controlled by antiviral mechanisms largely distinct from those active in the subsequent disseminated systemic infection [6]. To identify candidate antiviral factors and immune pathways solicited during the primary midgut infection, we fed mosquitoes on an ONNV-containing bloodmeal or control normal bloodmeal, purified RNA three days post-bloodmeal before virus dissemination to the systemic compartment, and transcriptionally profiled mRNA abundance by Illumina RNA sequencing (RNAseq). Virus bloodmeal titer was optimized to produce midgut infection rates of 100% among bloodfed mosquitoes, as previously determined [6], which augments experimental and statistical power. Although ONNV infection is limited to the midgut at 3 days (d) post-bloodmeal, we purified RNA from entire mosquitoes in order to also detect any potential systemic signaling prior to virus escape from the midgut.

**Fig. 1:**
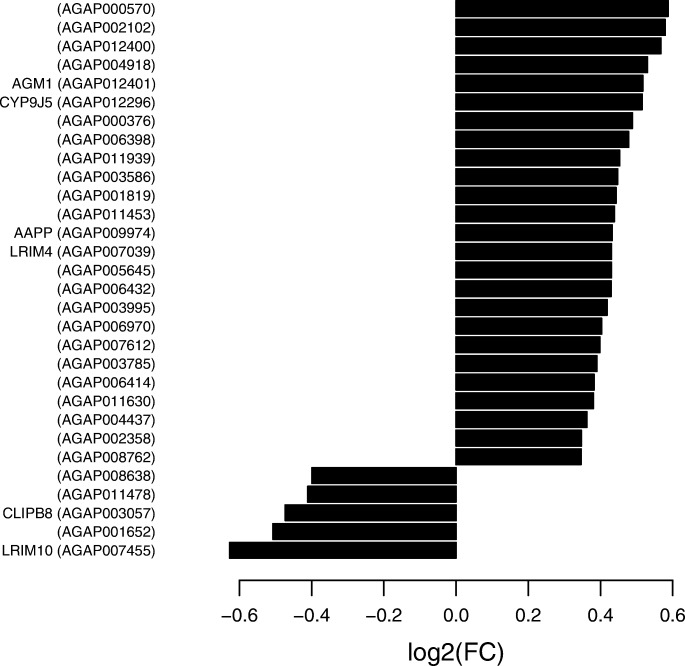
Thirty *A. coluzzii* genes are significantly regulated during the primary midgut infection with ONNV. Differential gene expression between ONNV-infected blood meal and non-infected bloodmeal was measured by RNAseq in pools of *A. coluzzii* mosquitoes 3 d after the bloodmeal, a time point when the infection is restricted to the midgut epithelium and not yet disseminated in the body compartment [6]. Histograms indicate fold change of transcript abundance (adjusted *p*-values, Additional File 1: Table S1). Gene identities are indicated by Vectorbase AGAP identifiers. The six genes with a gene name to the left of the AGAP identifiers have an annotated function, while the majority of ONNV-regulated genes are without known function, and bioinformatic functional prediction data is shown in Additional File 1: Table S1

Only 30 candidate genes were significantly regulated during ONNV primary infection of *A. coluzzii* as compared to mosquitoes fed a normal bloodmeal, generating a discrete and highly focused transcriptome response (Fig. 1; Additional file 1: Table S1, column F: ONNV log2FoldChange, column G: ONNV adjusted *p*-value). Of the 30 regulated candidates, just six have assigned gene names. Thus, the majority of ONNV-regulated genes carry little or no functional information, and are difficult to interpret. Functional analysis using Gene Ontology (GO) terms detected significant enrichment for predicted carbohydrate metabolic processes (GO:0005975, carbohydrate metabolic process, 201 annotated, 0.67 expected, 6 observed, *p* = 3.06e-05; Additional file 1: Table S1, column H: Associated GO-terms), suggesting that ONNV replication and/or the host immune response may impose metabolic costs on the mosquito host. The ONNV-regulated candidate genes include known or predicted immune-related genes LRIM4, LRIM10, CLIPB8, FREP63 and AGAP000376, although the different GO terms associated with immunity were not significantly enriched. The latter gene, AGAP000376, displays orthology with *Drosophila melanogaster* tsf1, a midgut immune factor in the Imd immune pathway (Additional file 2: Figure S1) [11–13].

### Comparison with other *Anopheles* transcriptional datasets

**Fig. 2:**
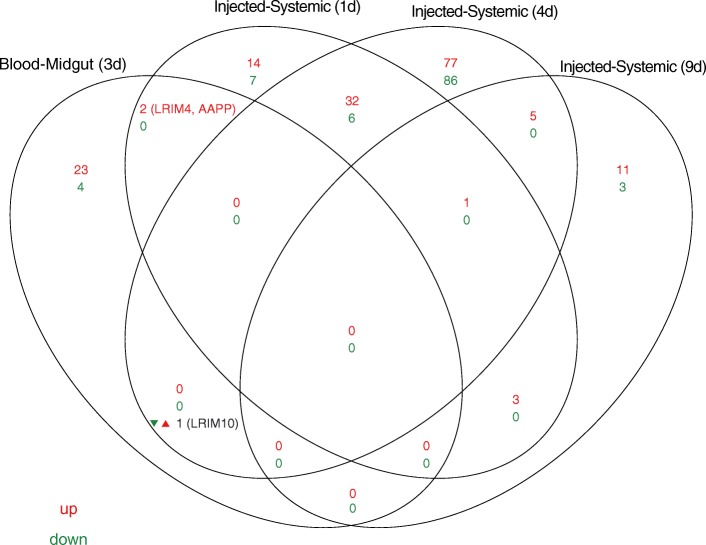
Comparison of ONNV regulated gene sets after bloodmeal or injection-induced infections of *A. coluzzii*. Venn diagram indicates overlap of ONNV-regulated genes in the primary midgut infection by bloodmeal (Blood-Midgut 3d, current study) and in the systemic infection by injection of virus, bypassing the midgut and without bloodmeal, at 1 d (Injected-Systemic 1d), 4 d (Injected-Systemic 4d), and 9 d (Injected-Systemic 9d) post-injection [10]. Number and names of overlapping genes upregulated (red), downregulated (green) and differently regulated (black) between studies are indicated. Three genes were regulated in both bloodmeal and injection-induced infections: LRIM4 and AAAP were upregulated in both conditions while LRIM10 was downregulated in blood-infected but upregulated in injection-infected mosquitoes

The current Illumina RNAseq dataset was compared to previously reported microarray gene expression data, also from *A. coluzzii,* infected by intra-thoracic injection with ONNV [10]. In that study, 211 transcripts were differentially regulated at four days post-injection. Only three genes overlap with the transcripts regulated by the normal bloodmeal-infection route used in our study (Fig. 2): LRIM4 (AGAP007039) and AAPP (AGAP009974) are upregulated both in blood-infected midgut and needle-infected mosquitoes at one day post-injection, while LRIM10 is downregulated in blood-infected midgut but was upregulated in needle-infected mosquitoes at four days post-injection.

The small number of overlapping regulated genes between the studies, and the opposite regulation of LRIM10, is consistent with the dichotomy in immune signaling previously described for the two compartments, that is, the primary midgut infection by bloodmeal, and the secondary disseminated systemic infection [6]. Needle infection is a model for the secondary disseminated viral infection, bypassing the primary midgut infection, including the processes of the MIB and MEB. After needle-infection, most cell types of the mosquito are infected rapidly, which could explain the large differential transcript set, while infection of the midgut epithelium by bloodmeal is kinetically different because of the retarding effect of the midgut infection barrier, and may even produce systemic signals that induce qualitatively different responses in the secondary disseminated infection [14]. However, an influence of technical differences between the studies for infection methods (bloodmeal without wounding, or needle infection with wounding), and transcript profiling platforms (RNAseq or microarray) also cannot be excluded.

We compared differential regulation induced by ONNV infection of the midgut with previously reported expression data from *A. coluzzii* infected with the midgut stage of *Plasmodium falciparum* [15]. Unfortunately, a comprehensive comparison is not possible, because the *Plasmodium* infection data are from 2006, and used a now-obsolete reference genome annotation that contained different gene models. Many gene IDs from the annotation used in the *Plasmodium* study were retired, some gene IDs were split, and many new gene IDs were created since then. Technical differences would also limit the value of completely remapping the 2006 microarray probe sequences to current candidate gene IDs, because the microarray coverage of transcripts was much lower than the current RNAseq coverage. Nevertheless, we remapped the recognizable gene ID matches to the current *A. gambiae* PEST strain genome (assembly AgamP4) in order to compare the two biological datasets to the extent possible.

Among the 30 current ONNV-regulated transcripts, a total of 12 gene IDs could be linked to old gene IDs from the *Plasmodium* dataset (Additional File 1: Table S1, column U: ID_Old-ENSANGT). All 12 shared transcripts were induced by ONNV infection (Additional file 3: Figure S2; Additional file 1: Table S1, column V: ONNV, 1 = induced, 0 = no change, − 1 = repressed). Seven of the transcripts were also induced by mosquito feeding on a viable but invasion-defective *P. falciparum* mutant (Additional file 1: Table S1, column Y: PfCTRP), while only four and three genes were regulated by midgut infection with *P. falciparum* and *P. berghei*, respectively (Additional file 1: Table S1, column W, PfGUT, column X, PbGUT). There is no clear pattern of gene function among the shared genes, but this is not surprising given the small numbers as well as the low level of gene functional annotation. A comprehensive comparison would require repeating the *Plasmodium* study using RNAseq analysis mapped to the current reference genome and gene IDs.

### Differential miRNA abundance during ONNV infection

The small RNA fraction was sequenced from the same above *A. coluzzii* samples fed on an ONNV-containing or normal bloodmeal, and was analyzed using miRDeep2. 118 known miRNAs were detected, and 182 potential novel miRNAs were predicted in *A. coluzzii* (Additional file 4: Table S2). These figures are similar to the total number of 256 known miRNAs in Drosophila (miRbase BDGP5.0).

Differential analysis of miRNA abundance after ONNV infection detected one miRNA (aga-chr2L_23370) that was regulated during the primary infection of *A. coluzzii* with ONNV (Additional file 5: Figure S3, Additional file 4: Table S2). We observe a log2 fold change of 1.949 of miRNA aga-chr2L_23370 in ONNV-infected mosquitoes as compared to normal bloodfed controls (adjusted *p* = 2.52e-04). This miRNA was identified in a recent catalog of *Anopheles* miRNAs [16]. That analysis identified four miRNAs significantly regulated by bloodmeal, and six by *P. berghei*-infected blood, but levels of aga-chr2L_23370 were not altered by any condition tested. These two sets of results suggest that neither viral nor *Plasmodium* infection exerts a large impact on *Anopheles* miRNA expression. However, one miRNA displayed a functional effect on the efficiency of *Plasmodium* development in *Anopheles* [17, 18].

Thus, to date ONNV infection is the only condition known to regulate the expression level of miRNA aga-chr2L_23370. The potential functional effect of miRNA aga-chr2L_23370 on the host response to ONNV infection, and viral replication, will require further work to understand.

### Correlation of miRNAs with regulation of mRNA expression

Sequence-based target prediction methods of miRNAs are based on the prediction of complementarity between the seed region of mature miRNAs (nucleotide positions 2–7) and the 3’ UTR region of messenger RNAs [19, 20]. A drawback of these methods is a high rate of false positive predictions due to the small size of the seed region of mature miRNAs [21]. Consequently, it has been recommended to filter these sequence based predictions by using the joint analysis of expression levels of miRNAs and mRNA targets [22].

In order to generate a large dataset to empirically test the correlation between miRNA expression and regulation of mRNAs, we silenced the key immune signaling nodes, Rel2 and Stat-A, with and without ONNV infection, to perturb large networks of gene expression in the signaling pathways Imd and JAK/STAT, respectively. These two immune pathways were chosen because we previously showed that their activity is required for full *A. coluzzii* antiviral protection from initial midgut ONNV infection [6]. Perturbing these pathways caused differential expression of a total of 11 miRNAs, with expression levels of five miRNAs altered by silencing of Rel2, four by silencing of Stat-A, and two by control treatment with dsRNA for LacZ (Additional file 4: Table S2). The behavior of these regulated miRNAs clustered according to the different experimental treatments (Additional file 6: Figure S4). Consistent with the results above indicating a small and focused ONNV-dependent regulation of abundance of mRNAs (Fig. 1) and miRNAs (Additional file 5: Figure S3, Additional file 4: Table S2), the hierarchical clustering of miRNA expression response was driven largely by the pathway silencing treatments, with little effect due to the presence or absence of ONNV infection.

**Fig. 3:**
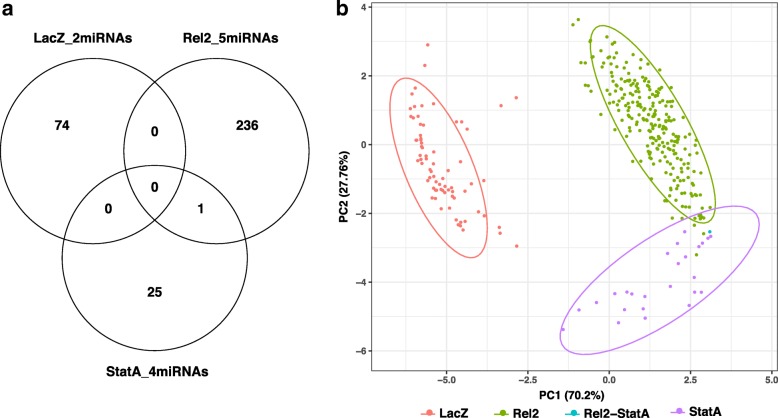
Correlation between miRNA target gene prediction and differential expression of predicted targets in *A. coluzzii*. **a**) Venn diagram of Pearson correlation between predicted miRNA target genes and their transcript abundance levels as measured by RNAseq. Silencing of the Imd pathway factor Rel2 significantly regulated five miRNAs (Rel2_5miRNAs), silencing the JAK/STAT factor Stat-A regulated four miRNAs (StatA_4miRNAs), and control treatment with LacZ dsRNA regulated two miRNAs (LacZ_2miRNAs)(Additional file 6: Figure S4). Diagram indicates the numbers of in silico predicted target genes whose expression was significantly anti-correlated with the abundance of the miRNAs. **b**) PCA analysis of the Pearson correlation matrix for the 11 regulated miRNAs and the 336 differentially expressed predicted target genes represented in the Venn diagram. Points indicate genes that were differentially expressed and also predicted as miRNA targets, color of points indicates the treatment that caused the differential gene expression. Predicted target genes filtered for anti-correlation of expression with the miRNAs cluster strongly according to the conditions that generated the expression differences of the miRNA-mRNA target pairs

Here, we use deep sequencing of mRNAs and miRNAs in the same samples in order to empirically test the accuracy of sequence-based miRNA target predictions, and to ascertain candidate miRNA-mRNA pairs potentially involved in *A. coluzzii* antiviral immunity. Co-expression levels were determined for miRNA-mRNA target pairs, under the general mechanistic model assuming that miRNAs repress the expression of mRNA from their target genes [23]. When miRNA-mRNA target pairs from sequence-based predictions were filtered for those having a significant negative correlation for expression, the number of potential target genes was 336, with just one gene (AGAP001650) potentially targeted by both Rel2 and Stat-A-induced miRNAs (Fig. 3a, Additional file 7: Table S3). Principal component analysis (PCA) of the Pearson correlation matrix for the 11 miRNAs and the 336 filtered candidate mRNA targets demonstrates a clear clustering of target genes of miRNAs induced under different treatments (Fig. 3b).

**Fig. 4:**
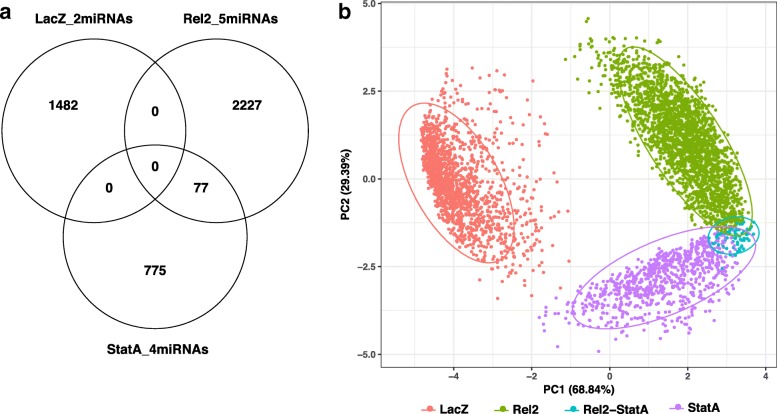
Genes with mRNA abundance significantly anti-correlated with the 11 regulated miRNAs. **a**) Similar to Fig. 3, but filtering mRNAs only for significant negative correlation of expression with the 11 induced miRNAs (Additional file 6: Figure S4), without filtering by computational target site prediction. Venn diagram of Pearson correlation between miRNAs and the differentially expressed genes as measured by RNAseq. **b**) PCA analysis of the differentially expressed genes represented in the Venn diagram based on Pearson correlation coefficients with miRNA expression levels. Other description as in Fig. 3

We further explored the discovery of candidate target genes for regulation by specific miRNAs by imposing only a significance threshold for anti-correlation between expression levels of miRNA-mRNA pairs, without in silico target site prediction. When filtered strictly by significant anti-correlation, the number of potential target genes of the 11 miRNAs increased more than 10-fold to 4614 mRNA candidates induced among the different treatment conditions. This result is consistent with observations that computational miRNA target site prediction can have limited accuracy [24], and in particular can under-predict functional miRNA-mRNA interactions [25]. In the current case, filtering only for anti-correlation of abundance, clear clustering of target genes according to treatment is again observed (Fig. 4a, Additional file 7: Table S3), but now with greater density of candidate target genes within the clusters (Fig. 4b).

These results strongly suggest that nine miRNAs are involved in inhibiting specific clusters of target genes of the Imd and JAK/STAT immune pathways previously implicated in *A. coluzzii* midgut antiviral protection to ONNV [6]. In addition, another cluster of genes responding to two miRNAs induced after treatment with control LacZ dsRNA appear to be involved in wounding and/or exposure to dsRNA. Moreover, the results suggest that computational target site prediction may under-predict target genes in this system, although it cannot be ruled out that the additional fraction of anti-correlated mRNAs not predicted as target sites may also include genes indirectly regulated by direct miRNA targets.

## Discussion

Here we show that ONNV infection of *Anopheles*, the natural vector of this virus, causes a highly focused and limited impact on the host transcriptome during the primary midgut blood-induced infection. The most striking observation is that just 30 host candidate genes and one miRNA were regulated by ONNV infection when controlled for the effect of the bloodmeal alone. The midgut epithelial cells are the first point of contact between host and pathogen at the portal of initial infection. The host genes regulated during this initial interaction are likely to be crucial for the subsequent infection, probably including both host genes deployed for antiviral defense as well as genes manipulated by the virus to facilitate infection. A majority of the ONNV-regulated candidate genes have unknown molecular function. Little is known about *Anopheles* interaction with viruses, and functional dissection of these candidates would likely generate novel biological insights into the host-virus interaction, and the structure of *Anopheles* immunity.

Two main categories of observed regulated candidate genes are associated with carbohydrate metabolism and immunity. A simple interpretation of the carbohydrate metabolism category could be that viral infection of the midgut epithelium imposes a metabolic burden on the infected cells because of the demands of viral replication, simultaneous with the high energetic demands of bloodmeal digestion. Induction of immune processes probably limits viral replication and consequent cytopathic effects, but also imposes the metabolic costs of immunity. The net host response probably balances the fitness costs of viral replication with the costs and benefits of digestion and immunity. Converting the bloodmeal to egg production is probably prioritized by the host because it is directly linked to host reproductive fitness. Conversely, viral fitness is linked not only to replication but ultimately to transmission, which requires a vector healthy enough to fly and bite a vertebrate host. This need should modulate overt arbovirus pathogenicity to the mosquito host.

Mosquitoes are persistently exposed in nature to members of the natural virome, including insect-specific viruses (ISVs) [26–29], which have probably been an important evolutionary force shaping mosquito antiviral immunity. In general, prevalent members of the natural virome should evolve towards lower pathogenicity in their habitual host lineages. Consistent with that expectation, a symbiotic *Anopheles* densovirus (studied for its potential use as a paratransgenesis tool) elicited low transcriptional response upon infection, and neither mosquito age at infection nor feeding regime affected viral titers [30, 31]. However, maintenance of bacterial microbiome commensals in the non-pathogenic commensal state requires active policing by basal host immunity [32]. By analogy, the maintenance of adapted ISVs as non-pathogenic may also result from a dialog with host immunity. Presumably, the same antiviral mechanisms used in basal maintenance are also deployed against arboviruses when encountered, which are often in the same families as members of the insect virome [29].

There was only one previous study of gene expression in *Anopheles* infected with ONNV, in our knowledge, which displayed little similarity of gene regulation to the current study [10]. The difference between the study results can likely be explained by the experimental designs. The previous study infected the hemocoel by injection of ONNV, thus bypassing the midgut epithelium and physiological influence of virus delivery in a bloodmeal. The *Anopheles* antiviral response to ONNV is highly compartmentalized, and involves the activity of distinct signaling pathways and molecules in the primary midgut infection as compared to the secondary disseminated infection [6].

There have been several transcriptomic studies of the *Aedes aegypti* mRNA response to virus infection. The results of one study [33] are not comparable to ours because tissues were analyzed 10 d after an infective bloodmeal, which detected mainly the secondary systemic dissemination into all tissues including midgut. Another study used needle infection of viruses for transcriptome discovery [34]. A recent study delivered chikungunya virus in an oral saline meal rather than bloodmeal, and detected 78 differentially regulated transcripts at 2 d post-infection [35].

Two studies with comparable experimental design to detect the primary midgut infection response were reported in *Ae. aegypti*. One study after a dengue virus oral bloodmeal detected 30–89 differential transcripts at, respectively, 1 d and 4 d post-infection [36]. More recently, analysis of the transcriptomic response of *Ae. aegypti* miRNAs [37] and mRNAs [38] after Zika virus infection detected 17 differentially regulated miRNAs, and 54 or 101 differentially expressed mRNAs at 2 d and 7d post-infection, respectively. The results of these two studies in *Ae. aegypti* both appear broadly similar to the current *Anopheles* and ONNV study in the magnitude of mRNA response to viral infection, while the *Ae. aegypti* miRNA response may appear more robust than the miRNA regulation in *Anopheles*.

A ternary immune complex protects *A. coluzzii* against *Plasmodium* infection, and is comprised of the leucine-rich repeat (LRR) immune factor LRIM1, either APL1A or APL1C, and a complement cofactor Tep1, Tep3 or Tep4 [39–41]. We previously found that activities of APL1A and APL1C are required for antiviral protection against ONNV midgut infection in *A. coluzzii*, but that the cofactors in the anti-*Plasmodium* response, LRIM1, Tep1, and Tep3, are not involved [6]. In the current study, we observe that two LRR proteins are regulated during ONNV infection. LRIM4 is upregulated whereas LRIM10 is downregulated, suggesting that LRIM4 could be a partner in a putative antiviral ternary complex in conjunction with APL1A or APL1C, and an unknown Tep factor.

We also identify the clip-domain serine protease CLIPB8 as a downregulated candidate gene during ONNV infection. CLIP proteins modulate diverse immune pathways in insects. CLIPB8 is a component of the *Anopheles* pro-phenoloxidase activation system [42], and is required for melanization of *P. berghei* parasites and Sephadex beads [43, 44]. The repression of CLIPB8 during ONNV infection raises the possibility that either the protective response of *Anopheles* to ONNV infection requires an inhibition of the melanization cascade, or that the virus suppresses the cascade as host manipulation. That possibility would be consistent with the observed decrease of melanization of *P. berghei* parasites in mosquitoes co-infected with ONNV [10].

The extensive small RNA sequence data in this study produced a comprehensive database of known and potential miRNAs in *A. coluzzii*. We identified one miRNA, aga-chr2L_23370, which is differentially regulated by ONNV expression. Characterizing this miRNA and its target genes could shed light on the RNA virus-*Anopheles* interaction. No previous miRNA studies have been performed on the *Anopheles* viral response to our knowledge. Multiple studies in *Aedes* or *Culex*, often cell lines, have identified miRNAs that respond to arbovirus infection, and can participate in antiviral protection [37, 45–51]. A comparable study in *Ae. aegypti* blood-infected with Zika virus detected ten miRNAs significantly regulated at 2 d post-bloodmeal as compared to normal bloodmeal [37].

Interestingly, nine *A. coluzzii* miRNAs were differentially regulated by silencing of the Imd and JAK/STAT immune signaling pathways (with two more miRNAs regulated by the dsLacZ control), suggesting an influence by miRNA expression upon mosquito basal immunity. Anti-correlation of these 11 miRNAs with transcriptome-wide mRNA abundance, either with or without miRNA target site prediction, identified defined clusters of regulated genes that plausibly respond to miRNA control. These results provide novel candidate miRNAs and target gene networks for the study of *Anopheles* immune and antiviral regulation.

## Conclusions

O’nyong nyong virus (ONNV) is the only virus known to be consistently transmitted by *Anopheles* mosquitoes. *Anopheles* are efficient vectors of human malaria, but it is unknown why they do not transmit viruses as efficiently as *Aedes* and *Culex* mosquitoes. Here, we identified the candidate genes that respond to ONNV infection in the mosquito stomach after virus infection. The cells of the stomach are the first site of antiviral defense, and early events there are likely to be crucial for the course of the subsequent infection. We found that the expression levels of only 30 candidate genes are changed by primary infection with ONNV. The function of most of the genes is unknown, but they probably include a combination of mosquito self-defense functions, and also mosquito genes that are manipulated by the virus in order to promote infection. Nevertheless, the vector must remain fit enough to fly in order to transmit the virus, suggesting that the virus may prioritize viral stealth and immune evasion rather than pathogenicity. Examination of the ONNV-response candidate genes to determine their functions is expected to generate novel insight into the mechanisms of virus-vector interaction.

## Methods

### Mosquito colonies

The *A. coluzzii* Ngousso strain was originally initiated with mosquitoes collected in Cameroon in January 2006, and belongs to the M molecular and Forest chromosomal forms [52]. Mosquitoes were reared under standard conditions at 26 °C and 80% relative humidity, with a 12 h light/dark cycle as previously described [41].

### Viruses and mosquito infection

The ONN-eGFP infectious clone of the ONNV SG650 strain [7] was kindly provided by Dr. Brian Foy, Colorado State University. ONNV SG650 was first isolated from human serum in Uganda in 1996. Infectious clone cDNA was linearized by *Not*I, and viral RNA was transcribed using T7 RNA polymerase (NEB), m7(5′)ppp(5′)A-Cap Analog (NEB) and rNTPs (Promega). Transcribed RNA was electroporated into BHK-21 cells. Virus was recovered after 72 h, and passaged once on *A. gambiae* 4a3a cells [53, 54] to increase titers.

Mosquitoes were infected by feeding on an infectious bloodmeal containing viral titers at the high end as found at the peak of clinical viremia, as previously described [6]. These conditions ensure that all mosquitoes are positive for virus infection at 3 d post-bloodmeal, which augments experimental and statistical power. Briefly, female mosquitoes deprived of sugar for 12 h were allowed to feed for 15 min through a Hemotek membrane covering a glass feeder containing the blood-virus mixture maintained at 37 °C. The infectious bloodmeal was composed of a virus suspension diluted (1:3) in washed rabbit erythrocytes from arterial blood, resuspended at 50% in dialyzed rabbit serum (Sigma R4505). ATP was added to a final concentration of 5 μM. Fully engorged females were transferred to small cardboard containers and maintained with free access to 10% sucrose at 28 ± 1 °C. Final bloodmeal titer fed to mosquitoes was between 1 to 3x10^7^pfu/ml as assessed by plaque assay.

### Double-stranded RNA synthesis and injection

Double-stranded RNAs were synthesized from PCR amplicons using the T7 Megascript Kit (Ambion) as described [6]. The sequences of primers used for synthesis of dsRNA templates are in Additional file 8: Table S4. For each targeted gene, 500 ng of dsRNA (up to 69.9 nl, depending on the concentration) was injected using glass capillary needles into the thorax of cold-anesthetized 1–2 d-old *A. coluzzii* females using a nano-injector (Nanoject II, Drummond Scientific). *dsLacZ* was used as control.

The efficiency of the gene silencing effect was monitored 2–3 d after dsRNA injection (at the time of infection) on a pool of 5 whole mosquitoes. One step RT-qPCR (Power SYBR Green RNA-to-Ct 1 Step Kit, Applied Biosystems) was performed following the manufacturer’s instructions. The sequences of primers used for gene knockdown verification are in Additional file 8: Table S4, the annealing step was performed at 60 °C.

### RNAseq and small RNA sequencing

12 mosquitoes per pool, in each of two biological replicate pools, were collected 3 d after infection by oral feeding with ONNV and homogenized in TriReagent (Sigma). The long RNA fraction (> 200 nt) and small RNA fractions (< 200 nt) were extracted from the same biological pool using Nucleospin miRNA (Macherey Nagel) using the Trizol protocol and submitted to a Bioanalyser (Agilent) for quality assessment.

For RNAseq, 10μg of RNA (> 200 nt fraction) was used to prepare directional libraries using the TruSeq Stranded mRNA Library sample prep kit with index sets A and B (Illumina), with the following protocol modifications. Chemical fragmentation of polyA RNA was done using Ambion reagent (AM8740), followed by purification on RNeasy columns (Qiagen, #74204). Phosphatase and PNK treatments were performed and followed by purification on RNeasy columns (Qiagen, #74204). The fragmented RNA was then ligated with 3′- and 5′- TruSeq adapters, as described in the original protocol. Synthesis of cDNA was performed by reverse transcription. The cDNA product was then specifically amplified by 11 cycles of PCR and products purified on Agencourt AMPure XP beads (Beckman Coulter Genomics, # A63881).

For small RNA sequencing, the 10- to 30-nt RNA fragments were purified from the small RNA fraction (< 200 nt) on a 15% urea PAGE (BioRad). Small RNA libraries were then prepared using TruSeq Small RNA Library reagents (Illumina). The purified small RNAs were ligated with 3’ RNA adaptor (RA3 5’-TGGAATTCTCGGGTGCCAAGG). The 5’ RNA adaptor (RA5 5’-GUUCAGAGUUCUACAGUCCGACGAUC) was then ligated to the 5′ phosphate end of the small RNAs. Reverse transcription was done to convert RNA to cDNA, which was then selectively enriched by 11 cycles of PCR. The PCR products were purified on 5% PAGE (BioRad) and checked on a Bioanalyser DNA1000 chip (Agilent).

The resulting libraries were quality-controlled on a Bioanalyser DNA1000 chip (Agilent). Libraries were sequenced using the Illumina Hiseq 2000 in a multiplexed 51 + 7 bases single read using a TruSeq SR cluster kit v3 cBot HS, and a TruSeq SBS kit v3 HS 50 cycles (Illumina). Primary analysis of the sequences was performed with Casava software (v1.7 Illumina). Library preparation and sequencing was performed by the Transcriptomics and Epigenomics Core Facility of Institut Pasteur.

### Analysis of RNAseq sequencing data

Raw fastq files were clipped of their adaptor and filtered for quality using Cutadapt [55]. Sequence reads were then mapped against the *A. gambiae* PEST strain genome (assembly AgamP4) using BWA mem version 0.7.7 with default parameters [56]. Read counting in genes/features was done with HTSeq-count version 0.6.1 [57] and the AgamP4.3 gene set. Differential gene expression analysis was done under R version 3.3.1 [58] with the package DESeq2 version 1.14.1 [59] The raw *p*-values were adjusted for multiple testing according to the Benjamini and Hochberg procedure [60] and only the genes with an adjusted p-values under 0.05 were considered as differentially expressed. Venn Diagrams were drawn using the R package VennDiagram version 1.6.0, and GO term analysis was performed using the R package topGO [61].

### Analysis of small RNA sequencing data

Raw fastq files were clipped of their adaptor and filtered for quality with Cutadapt [55]. Next, a database of miRNA was build using reference miRNA from miRBase [62] and from the literature [16, 63]. For this purpose, genomic coordinates of reference miRNAs in gff format were integrated with bedtools v2.26 [64] in order to generate a non-redundant set of 167 *A. coluzzii* miRNA genes. Genomic coordinates of miRNA genes are based on *A. gambiae* PEST strain genome (assembly AgamP3).

The miRNA database was complemented using our small RNA datasets to predict potential novel miRNA using miRdeep2 version 2.0.0.7 [65] (miRNA_ONY_pre.fasta + mature), which yielded a prediction of 300 miRNAs, of which 118 were reference miRNAs and 182 were novel miRNAs detected in the present study (Additional file 4: Table S2, miRNA databases).

The quantifier.pl module of miRdeep2 was used to map filtered and clipped small RNA sequence reads over reference and novel miRNAs, quantifying the number of reads falling into an interval 2 nt upstream and 5 nt downstream of the mature/star sequence. Raw counts were analyzed for differential expression using DEseq 2 [59] using same procedure followed with RNAseq data above.

Sequence-based prediction of target genes of reference and new miRNAs were performed with MiRanda [66]. Pairwise comparison between log 10 transformed normalized expression matrices of mRNAs and miRNAs were performed using Pearson correlation with rcorr package in R version 3.3.1.

## Additional files

Additional file 1: **Table S1.** *A. coluzzii* mRNAs differentially expressed 3 d after ONNV infection by bloodmeal as compared to control mosquitoes fed a normal bloodmeal. Table lists differential regulation data for the 30 ONNV-regulated genes shown in Fig. 1, including bioinformatically predicted functional information where available. (XLSX 13 kb)

Additional file 2: **Figure S1.** Vector base ortholog representation of AGAP000376. (PDF 13 kb)

Additional file 3: **Figure S2.** Venn diagram of differentially expressed transcripts in *A. coluzzii* infected with ONNV or *Plasmodium*. Transcriptional response of *A. coluzzii* to ONNV infection 3 d post-bloodmeal as measured by RNAseq in the current study (ONNV-Gut) is compared to a published study of *A. coluzzii* response to *Plasmodium* infection as measured by microarray (Dong et al., 2006). Compared conditions were transcripts differentially expressed from *P. falciparum*–infected midgut (Pb-Gut), *P. berghei*-infected midgut (Pb-Gut), or midgut after a bloodmeal containing an invasion-incompetent mutant of *P. falciparum* (Pf-CTRP). (PDF 181 kb)

Additional file 4: **Table S2.** *A. coluzzii* miRNAs and regulation by treatments. Table includes novel and known miRNAs, sequences, and differential regulation data after dsRNA treatments, in mosquitoes with and without ONNV infection. Genomic coordinates of miRNA genes are based on *A. gambiae* PEST strain genome (assembly AgamP3). (XLSX 59 kb)

Additional file 5: **Figure S3.** Venn diagram of *A. coluzzii* differentially expressed miRNAs during ONNV infection. Mosquitoes were either not treated with dsRNA (no dsRNA) or were treated before bloodfeeding, with or without ONNV, with dsRNA for control LacZ (dsLacZ treatment), for Imd pathway factor Rel2 (dsRel2 treatment), or for JAK/STAT pathway factor Stat-A (dsStat−A treatment). Name and number of differentially expressed miRNAs are indicated. Details on miRNAs are in Additional File 4: Table S2. (PDF 147 kb)

Additional file 6: **Figure S4.** Hierarchical clustering of 11 *A. coluzzii* miRNAs differentially expressed among treatments. Treatments are indicated on the right vertical axis. Mosquitoes were either not treated with dsRNA (No-dsRNA) or were treated before bloodfeeding with dsRNA for control LacZ (dsLacZ), for Imd pathway factor Rel2 (dsRel2), or for JAK/STAT pathway factor Stat-A (dsStat−A). Bloodfeeding was either without ONNV (Non-infected) or with ONNV (Infected). Names of miRNAs are indicated on the x-axis. Details on miRNAs are in Additional File 4: Table S2. (PDF 292 kb)

Additional file 7: **Table S3.** *A. coluzzii* miRNA-mRNA target gene pairs by sequence-based target prediction and anti-correlation with mRNA expression levels. Table indicates complete transcript table with miRNA data. (XLSX 6821 kb)

Additional file 8: **Table S4.** Primer list. Primers used for synthesis of double-stranded RNAs (prefix T7, T7 RNA polymerase promoter underlined) or qPCR analysis (prefix q) of target genes. Final suffix indicates forward, F, or reverse, R, sense of primers. (DOCX 13 kb)

## Abbreviations

CHIKV: Chikungunya virus
d: Days
GO: Gene Ontology
LRR: Leucine-rich repeat
MEB: Midgut escape barrier
MIB: Midgut infection barrier
miRNA: Micro RNA
mRNA: Messenger RNA
ONNV: O’nyong nyong virus
PCA: Principal component analysis
RNAseq: Illumina RNA sequencing
