# Supplementary material for: Highly focused transcriptional response of Anopheles coluzzii to O’nyong nyong arbovirus during the primary midgut infection
Source: BMC Genomics. 2018 Jul 9;19:526. doi: 10.1186/s12864-018-4918-0 (PMC6038350; doi:10.1186/s12864-018-4918-0)
Supplement: Supplementary file 2 — Figure S1. Vector base ortholog representation of AGAP000376. (PDF 13 kb) [file 12864_2018_4918_MOESM2_ESM.pdf]

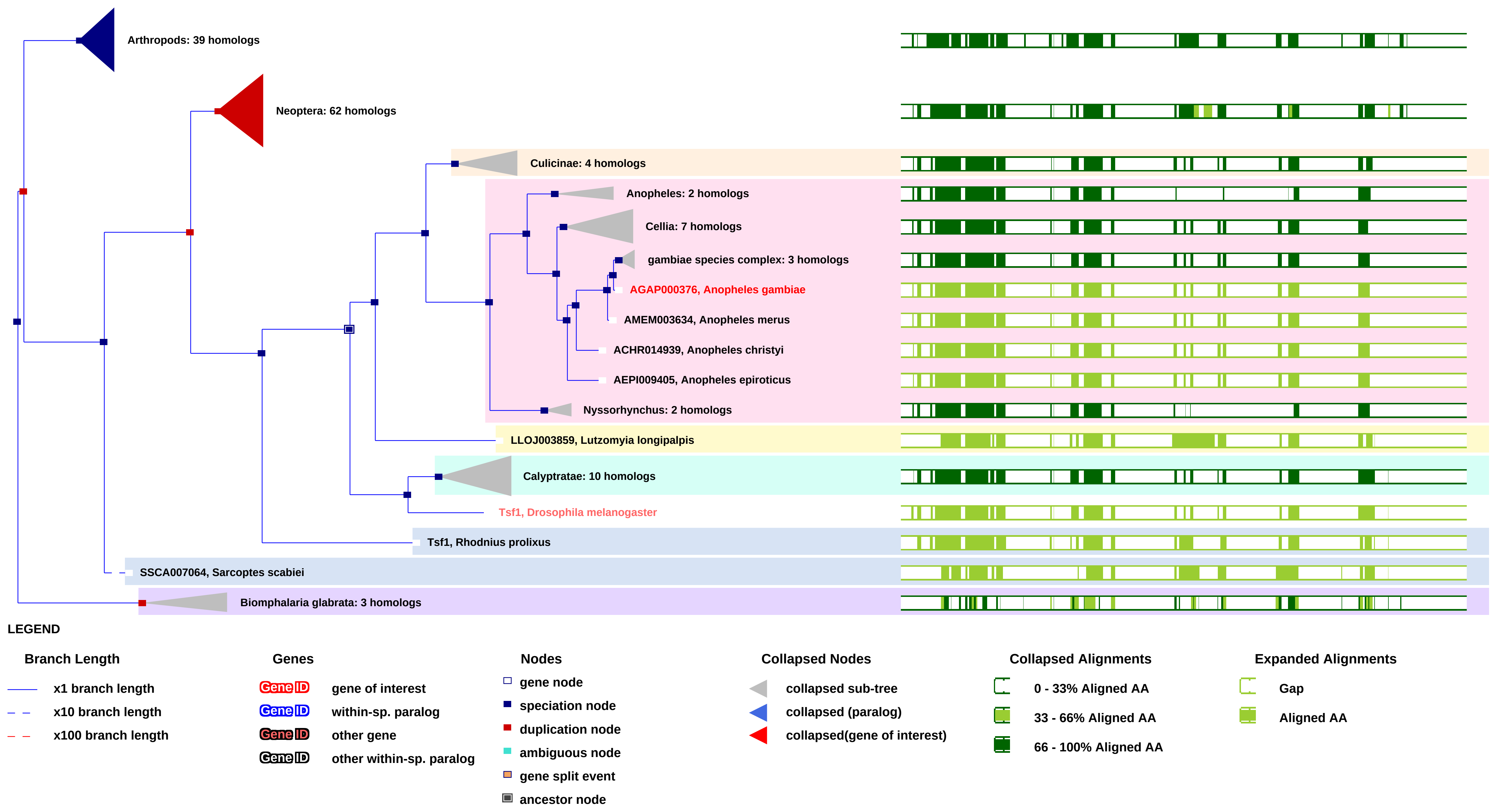

Arthropods: 39 homologs
Neoptera: 62 homologs
Culicinae: 4 homologs
Anopheles: 2 homologs
Cellia: 7 homologs
gambiae species complex: 3 homologs
AGAP000376, Anopheles gambiae
AMEM003634, Anopheles merus
ACHR014939, Anopheles christyi
AEPI009405, Anopheles epiroticus
Nyssorhynchus: 2 homologs
LLOJ003859, Lutzomyia longipalpis
Calyptratae: 10 homologs
Tsf1, Drosophila melanogaster
Tsf1, Rhodnius prolixus
SSCA007064, Sarcoptes scabiei
Biomphalaria glabrata: 3 homologs
LEGEND
Branch Length
x1 branch length
x10 branch length
x100 branch length
Genes
Gene ID
gene of interest
Gene ID
within-sp. paralog
Gene ID
other gene
Gene ID
other within-sp. paralog
Nodes
gene node
speciation node
duplication node
ambiguous node
gene split event
ancestor node
Collapsed Nodes
collapsed sub-tree
collapsed (paralog)
collapsed(gene of interest)
Collapsed Alignments
0 - 33% Aligned AA
33 - 66% Aligned AA
66 - 100% Aligned AA
Expanded Alignments
Gap
Aligned AA
