# Supplementary figures and images for: Highly focused transcriptional response of Anopheles coluzzii to O’nyong nyong arbovirus during the primary midgut infection
Source: BMC Genomics. 2018 Jul 9;19:526. doi: 10.1186/s12864-018-4918-0 (PMC6038350; doi:10.1186/s12864-018-4918-0)

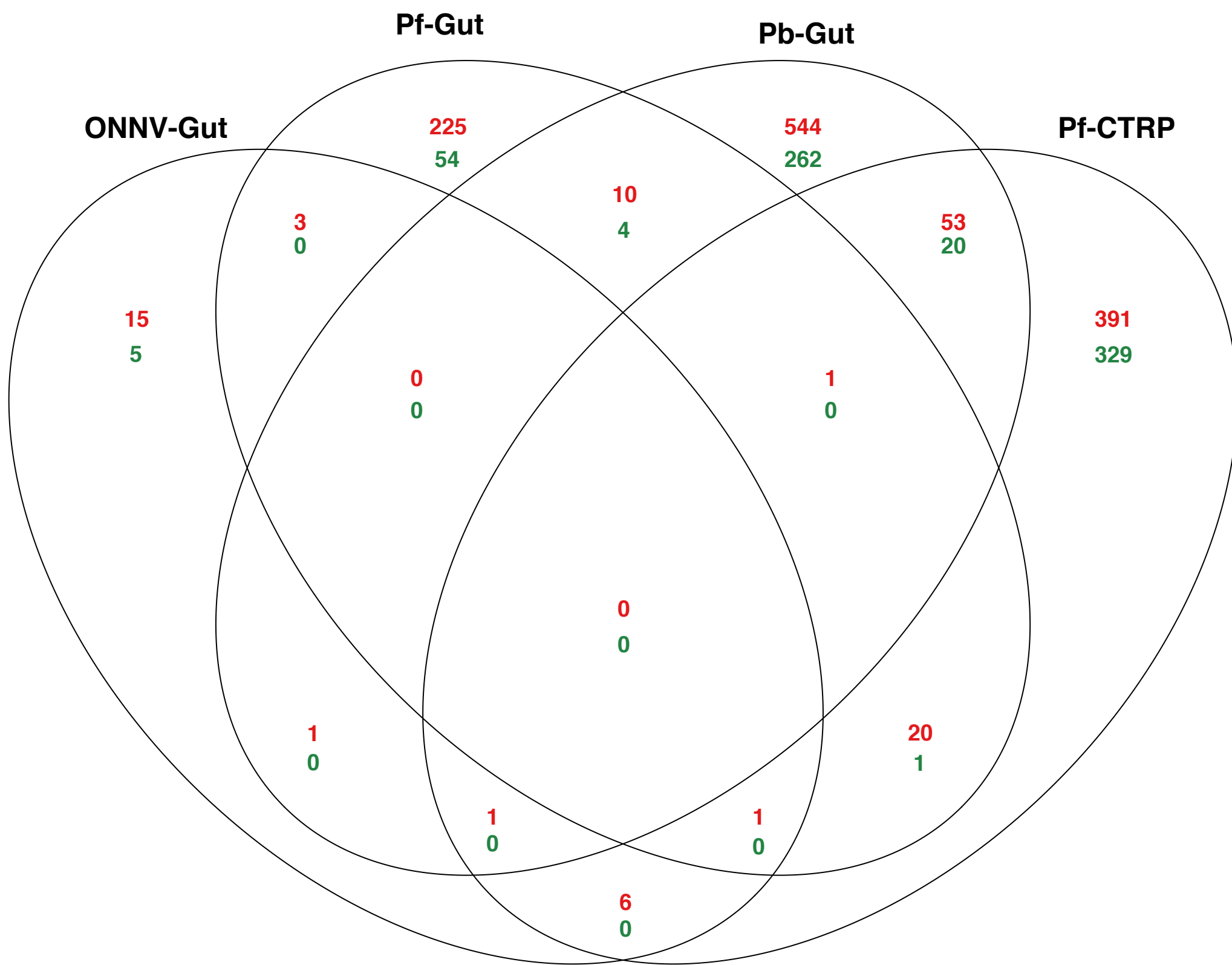

up

down

Supplement: Supplementary file 3 — Figure S2. Venn diagram of differentially expressed transcripts in A. coluzzii infected with ONNV or Plasmodium. Transcriptional response of A. coluzzii to ONNV infection 3 d post-bloodmeal as measured by RNAseq in the current study (ONNV-Gut) is compared to a published study of A. coluzzii response to Plasmodium infection as measured by microarray (Dong et al., 2006). Compared conditions were transcripts differentially expressed from P. falciparum–infected midgut (Pb-Gut), P. berghei-infected midgut (Pb-Gut), or midgut after a bloodmeal containing an invasion-incompetent mutant of P. falciparum (Pf-CTRP). (PDF 181 kb) [file 12864_2018_4918_MOESM3_ESM.pdf]

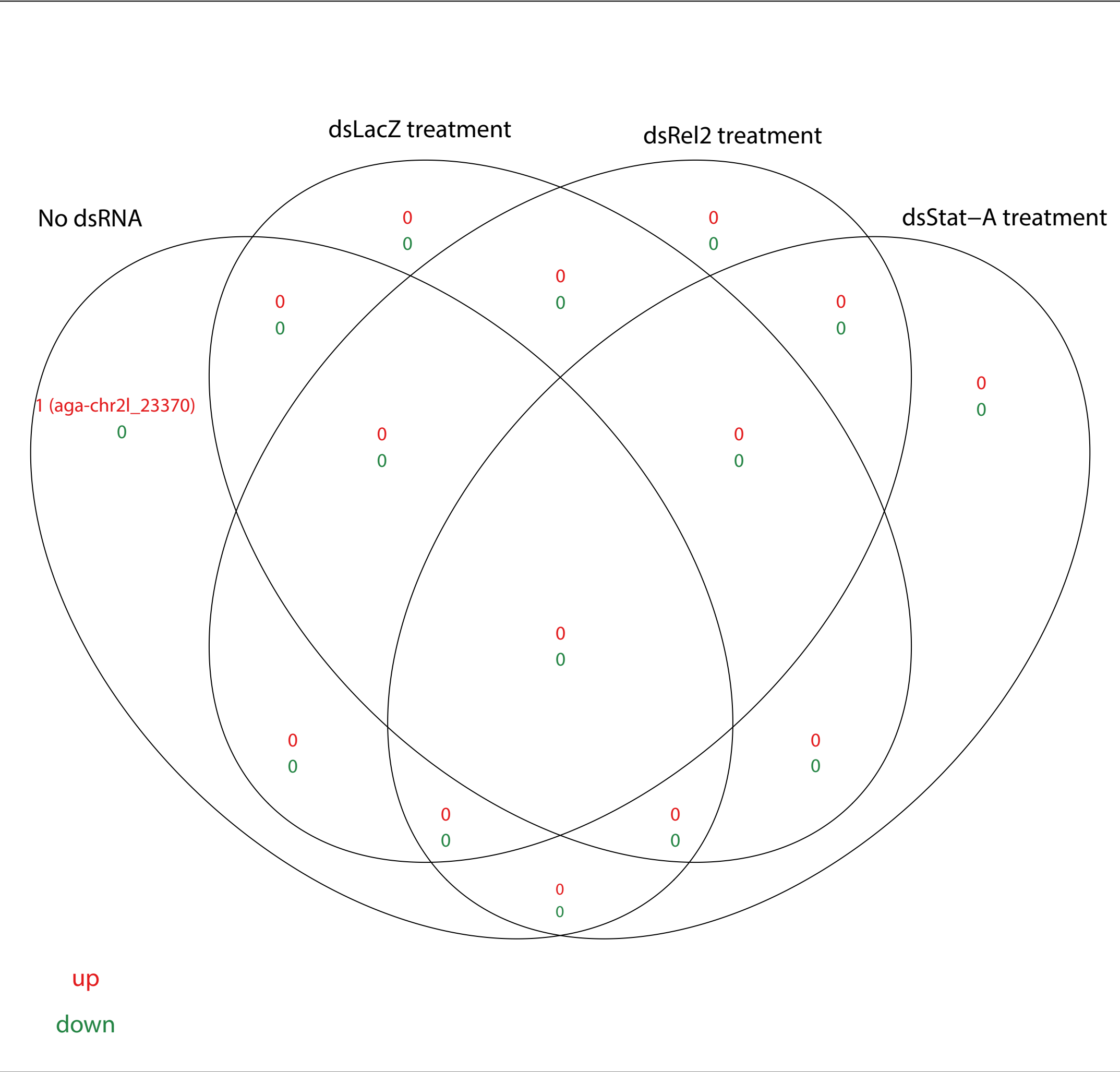

Supplement: Supplementary file 5 — Figure S3. Venn diagram of A. coluzzii differentially expressed miRNAs during ONNV infection. Mosquitoes were either not treated with dsRNA (no dsRNA) or were treated before bloodfeeding, with or without ONNV, with dsRNA for control LacZ (dsLacZ treatment), for Imd pathway factor Rel2 (dsRel2 treatment), or for JAK/STAT pathway factor Stat-A (dsStat−A treatment). Name and number of differentially expressed miRNAs are indicated. Details on miRNAs are in Additional File 4: Table S2. (PDF 147 kb) [file 12864_2018_4918_MOESM5_ESM.pdf]

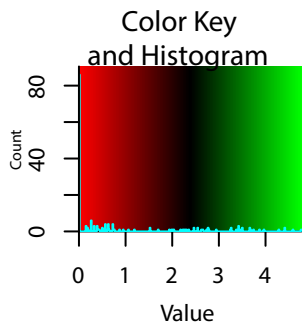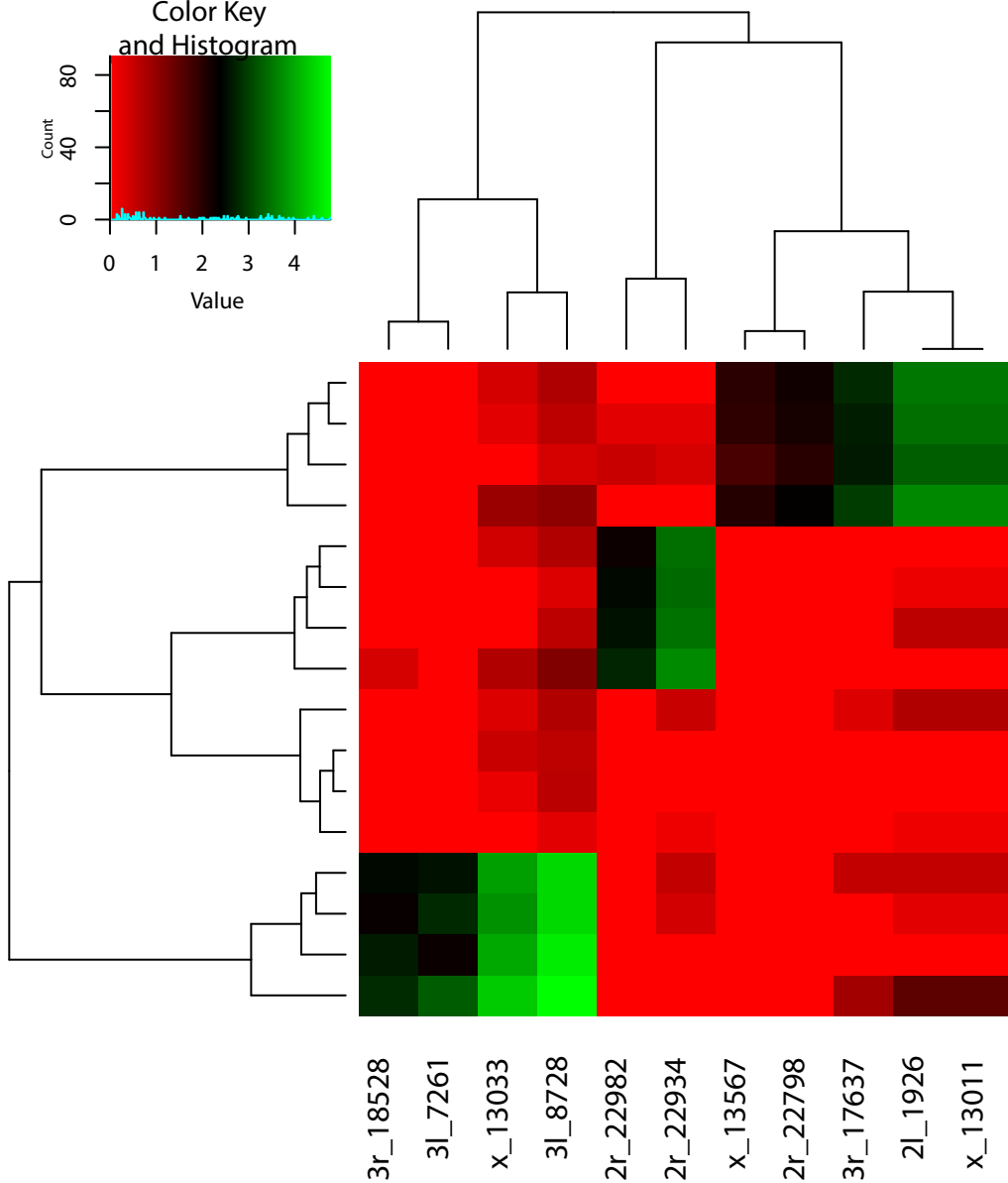

Supplement: Supplementary file 6 — Figure S4. Hierarchical clustering of 11 A. coluzzii miRNAs differentially expressed among treatments. Treatments are indicated on the right vertical axis. Mosquitoes were either not treated with dsRNA (No-dsRNA) or were treated before bloodfeeding with dsRNA for control LacZ (dsLacZ), for Imd pathway factor Rel2 (dsRel2), or for JAK/STAT pathway factor Stat-A (dsStat−A). Bloodfeeding was either without ONNV (Non-infected) or with ONNV (Infected). Names of miRNAs are indicated on the x-axis. Details on miRNAs are in Additional File 4: Table S2. (PDF 292 kb) [file 12864_2018_4918_MOESM6_ESM.pdf]
