# Supplementary material for: Highly focused transcriptional response of Anopheles coluzzii to O’nyong nyong arbovirus during the primary midgut infection
Source: BMC Genomics. 2018 Jul 9;19:526. doi: 10.1186/s12864-018-4918-0 (PMC6038350; doi:10.1186/s12864-018-4918-0)
Supplement: Supplementary file 8 — Table S4. Primer list. Primers used for synthesis of double-stranded RNAs (prefix T7, T7 RNA polymerase promoter underlined) or qPCR analysis (prefix q) of target genes. Final suffix indicates forward, F, or reverse, R, sense of primers. (DOCX 13 kb) [file 12864_2018_4918_MOESM8_ESM.docx]

| T7-LacZ-F | TAATACGACTCACTATAGGGGTCGCCAGCGGCACCGCGCCTTTC |
| --- | --- |
| T7-LacZ-R | TAATACGACTCACTATAGGGCCGGTAGCCAGCGCGGATCATCGG |
| T7-Rel2-F | TAATACGACTCACTATAGGGCAACAGCAGCAACAACATC |
| T7-Rel2-R | TAATACGACTCACTATAGGGCACAGGCACACCTGATTGA |
| T7-StatA-F | TAATACGACTCACTATAGGCCGGAGAGCAACTTCACGAT |
| T7-StatA-R | TAATACGACTCACTATAGGGATGAACGTGTTGTAATGAGC |
| qRel2-F | CGGGCAGAGGGAAGCAT |
| qRel-2-R | AGGCCCGCTCACCGTT |
| qStatA-F | TACAACGAAACGACCAAGCA |
| qStatA-R | GGTCCATACCGAAAAGACGA |
| qS7-F | AGAACCAGCAGACCACCATC |
| qS7-R | GCTGCAAACTTCGGCTATTC |

**Table S4.** Primers used for synthesis of double-stranded RNAs (prefix T7, T7 RNA polymerase promoter underlined) of target genes. Final suffix indicates forward, F, or reverse, R, sense of primers.
